# Advances in transport and toxicity of nanoparticles in plants

**DOI:** 10.1186/s12951-023-01830-5

**Published:** 2023-03-02

**Authors:** Mingyang Gao, Jia Chang, Zhongtang Wang, Hongyan Zhang, Tian Wang

**Affiliations:** Key Laboratory of Food Nutrition and Safety of Shandong Normal University, College of Life Sciences, Shandong Normal University, Jinan, 250014 People’s Republic of China

**Keywords:** Nanoparticles, Absorb, Transport, Phytotoxicity, Plants

## Abstract

In recent years, the rapid development of nanotechnology has made significant impacts on the industry. With the wide application of nanotechnology, nanoparticles (NPs) are inevitably released into the environment, and their fate, behavior and toxicity are indeterminate. Studies have indicated that NPs can be absorbed, transported and accumulated by terrestrial plants. The presence of NPs in certain edible plants may decrease harvests and threaten human health. Understanding the transport and toxicity of NPs in plants is the basis for risk assessment. In this review, we summarize the transportation of four types of NPs in terrestrial plants, and the phytotoxicity induced by NPs, including their impacts on plant growth and cell structure, and the underlying mechanisms such as inducing oxidative stress response, and causing genotoxic damage. We expect to provide reference for future research on the effects of NPs on plants.

**Graphical Abstract:**

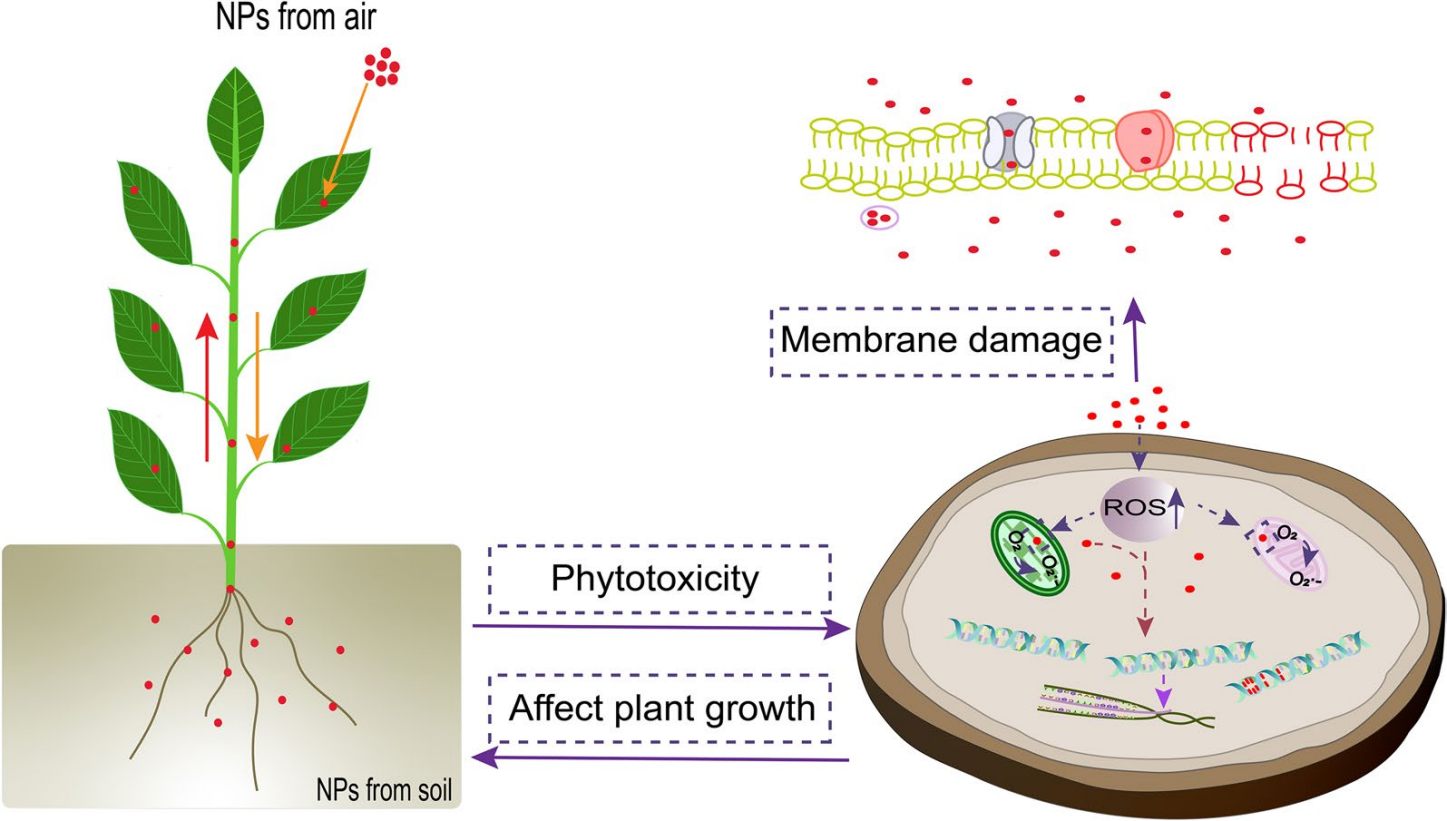

## Introduction

Nanoparticles (NPs) are a wide range of materials, at least one dimension less than 100 nm (nm) [1]. They have unique physicochemical properties compared with their bulk forms [2], which are normally caused by the subsize, chemical composition, surface structure, appearance and aggregation [3]. For this reason, NPs have made great achievements in a wide range of applications, including energy production, environmental remediation, and the food industry [4]. The increasing consumption of NPs inevitably enhances the chances of release into the environment during the process of production, recycling and waste disposal, resulting in the NPs accumulation in ecosystems [5].

It has been suggested that sewage treatment plants have become the main source of NPs in the soil, since the current sludge treatment process cannot effectively remove them, which leads to NPs existing in sludge and the application of sludge as biosolids in soil results in the exposure of NPs in soil [6]. Long-term repeated addition of biosolids to soil or application of nano-agriculture and soil remediation technology can lead to NPs accumulation for a long time and exposed to plants [7]. As a primary trophic level, plants are essential components of the ecosystems, representing the key elements in the food chain and providing food sources for human beings and animals [8]. With the development of nanotechnology and application of nanomaterials, NPs may be exposed to plants for a long time, posing a negative impact on plant growth, and may even be exposed to human body through food chain. Studies have shown that the cracks generated during lateral root formation may be the way for plants to absorb NPs from soil [9]. Stomata has also been proved to be an important way for plant leaves to absorb NPs, which is much larger than the aperture of biological barriers such as cell walls, providing more possibilities for absorption [10]. It means that perhaps NPs can be absorbed by plants trough down-to-up or up-to-down pathways. Some NPs have positive effects such as improving plant growth and increasing crop production when proper concentrations are used. However, more adverse effects of NPs have been reported including the inhibition of seed germination, the reduction of photosynthesis and disruption in plant root [11]. Researchers evaluated the exposure of micro and nano plastics in edible vegetables by scanning electron microscopy (SEM) combined with an X energy dispersion detector. The results showed that the levels of plastics distributed in carrots, lettuce, purslane, pear and apples were 101950 ± 44368, 50,550 ± 25,011, 126150 ± 80,715, 189550 ± 105558, and 195500 ± 128687 particles per gram, respectively [12]. Inductively coupled plasma mass spectrometry (ICP-MS) was used to quantify the accumulation of gold NPs. And according to different exposure methods (drop-cast and aerosol method) and different shapes of Au NPs (sphere, cube, rhombic dodecahedral, rod morphologies), the lowest recovery rate of Au NPs in leaves was about 27.9%, the highest was about 64.9%, and NPs could be transported to roots. The lowest recovery rate of Au NPs in roots was 7.3%, and the highest was 49.3% [13]. Quantitative results of NPs in plants indicated that the exposed NPs could be absorbed and transported by plants. At present, dozens of species have been proved to absorb NPs, including wheat, rice, maize, tomato, cucumber, lettuce, radish and peanut [14–21]. After being absorbed by plants, NPs can migrate to other tissues through the vascular system and pose cytotoxic and genotoxic effect on plants, including oxidative stress, antioxidant enzyme activity affection, and micronucleus and chromosome aberration index enhancement, consequently, affecting plant seed germination and root elongation [22, 23]. In recent years, the toxicity of NPs has attracted wide attention. Plant exposure to NPs may lead to high-level enrichment and migration to the food web, posing potential risks to human health [24]. A well understanding of the transportation of NPs on plants is the premise of risk evaluation. Moreover, the accumulation of NPs has adverse effects on plant development and the uptake of nutrients, resulting in reduce crop production [7, 25]. Among the studies on the interaction between NPs and plants, more studies reported their negative effects on plant growth. It is important to summarize the absorption and transport of NPs in plants and the effect of NPs on plants for future research and prevention of NPs pollution.

In this review, we describe the uptake and translocation of NPs in plants. The impacts of NPs on plants are also summarized in detail. The transport and toxicity mechanism of NPs is discussed to get a better understanding of interplay between NPs and plants.

### Transportation and distribution of NPs

To understand the absorption and transportation of NPs in plants can help interpret the toxicological data. Detection and characterization of NPs are particularly important in studying the absorption and transport of NPs in plants. At present, there are more than 20 kinds of NPs detection methods. Characterization of NPs to understand the distribution, stability, and structure of NPs is usually the first step. Transmission electron microscope (TEM), Raman spectrum (RS), X-Ray Diffraction (XRD) and dynamic light scattering (DLS) were used for characterization. Yang et al. measured the size of CeO_2_ NPs dispersed in deionized water by TEM, and the size and crystal structure of CeO_2_ NPs in dry powder samples by XRD [26]. DLS and laser confocal RS were used to measure the particle size distribution and molecular structure of PS-NPs [27]. The NPs were detected to understand the absorption and transportation mechanism of NPs in plants. Since the properties of NPs are different, and the plant components are complex, the appropriate methods for detecting NPs are limited. In previous studies, the most widely used methods to study the transport and distribution of metal and metal oxide NPs in plants are SEM and TEM that help observe the NPs in plant tissues, or X-ray spectra analysis to measure the metal elements in plants [28, 29]. Stegemeier et al. used μ-X ray fluorescence (μ-XRF) based on synchrotron to observe the distribution of Ag NPs (13.4 ± 2.9 nm) in duckweed roots. The map showed that Ag distributed throughout the root tip, especially in the meristem [30]. The content of Cu in rice roots exposed to CuO NPs was detected by inductively coupled plasma optical emission spectrometer (ICP-OES), and it was found that the content of Cu increased significantly. u-XRF analysis showed that Cu mainly accumulated in the white rice layer [31]. Different from metal-based NPs, carbon and plastic NPs in plants are not easy to be detected by elemental analysis, they are more commonly observed by SEM, TEM or fluorescent microscope after modifying fluorescent dyes [32–34]. Carbon dots (CDs) have photoluminescence characteristics, and after being exposed to tobacco cells, they could be monitored by fluorescence confocal microscope and the uptake of CDs might be evaluated by fluorescence analysis [35]. The imaging of laser confocal scanning microscope and SEM confirmed that polystyrene NPs (PS-NPs) could be absorbed by wheat roots and transported to shoots [36]. Although it is difficult to quantify plastic NPs compared with metal-based NPs, researchers have developed pyrolysis gas chromatography-mass spectrometry (GC–MS) for the quantification of plastic NPs in plants. The validity and application prospect of the method were verified by ICP-MS [37]. Compared with carbon NPs and plastic NPs, metal elements are easier to be detected by technical methods, so there are more ways to detect the absorption and distribution of metal NPs in plants. Besides the commonly used microscope and spectrum technology, there are more studies on quantitative detection based on IPC technology. Unfortunately, due to the complexity of plant sample matrix, the detection of carbon NPs and plastic NPs in plants mostly focuses on the application of imaging technology [38].

**Fig. 1:**
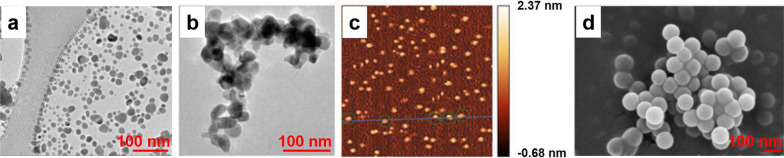
Characterization of NPs. **a** Metal NPs: 10 nm silver NPs [22]; (**b**) Metal oxide NPs: 79.6 ± 5 nm zinc oxide NPs [40]; (**c**) Carbon-based NPs: 2 nm CDs [41]; (**d**) 100 nm PS-NPs [42]. *CDs* carbon dots, *PS-NPs* polystyrene nanoplastics

The diversification of detection methods has laid a solid foundation for exploring the absorption and distribution of NPs in plants. According to a recent study, NPs could be absorbed by plants directly from the soil, penetrated plant tissues and migrated to different regions of plants, either through diffusion or endocytosis mechanisms [39]. Here, we summarize the uptake and transportation of four kinds of NPs in plants, including metal NPs, metal oxide NPs, carbon-based NPs and nanoplastics (Fig. 1).

### Transportation and distribution of metal NPs in plants

**Fig. 2:**
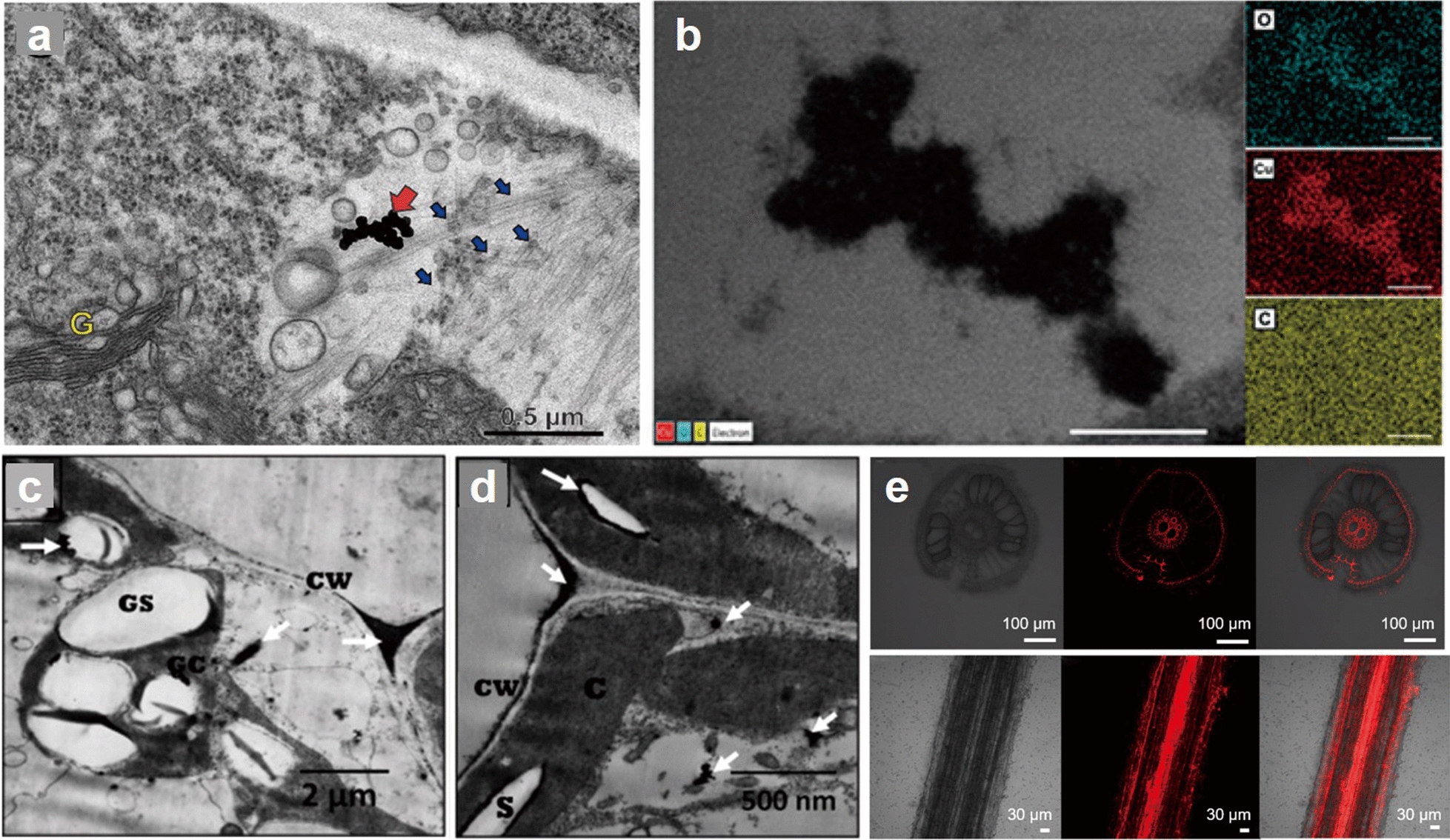
Transport and distribution of NPs in plants. **a** TEM image of Ag NPs in onion cells. Red arrows indicate NPs and blue arrows indicate microtubules [43]; (**b**) EDX mapping of wheat cells exposed to CuO NPs [19]; (**c**–**d**) Localization of GNS in leaves of eggplant and pepper (*C* elliptic chloroplasts, *CW* cell wall, *GC* giant chloroplasts, *GS* giant starch grains) [46]; (**e**) Transverse (upper) and longitudinal (lower) confocal images of rice roots exposed to plastic NPs [47]. *TEM* transmission electron microscopy, *EDX* energy-dispersive X-ray spectroscopy, *GNS* graphene nanosheets

**Fig. 3:**
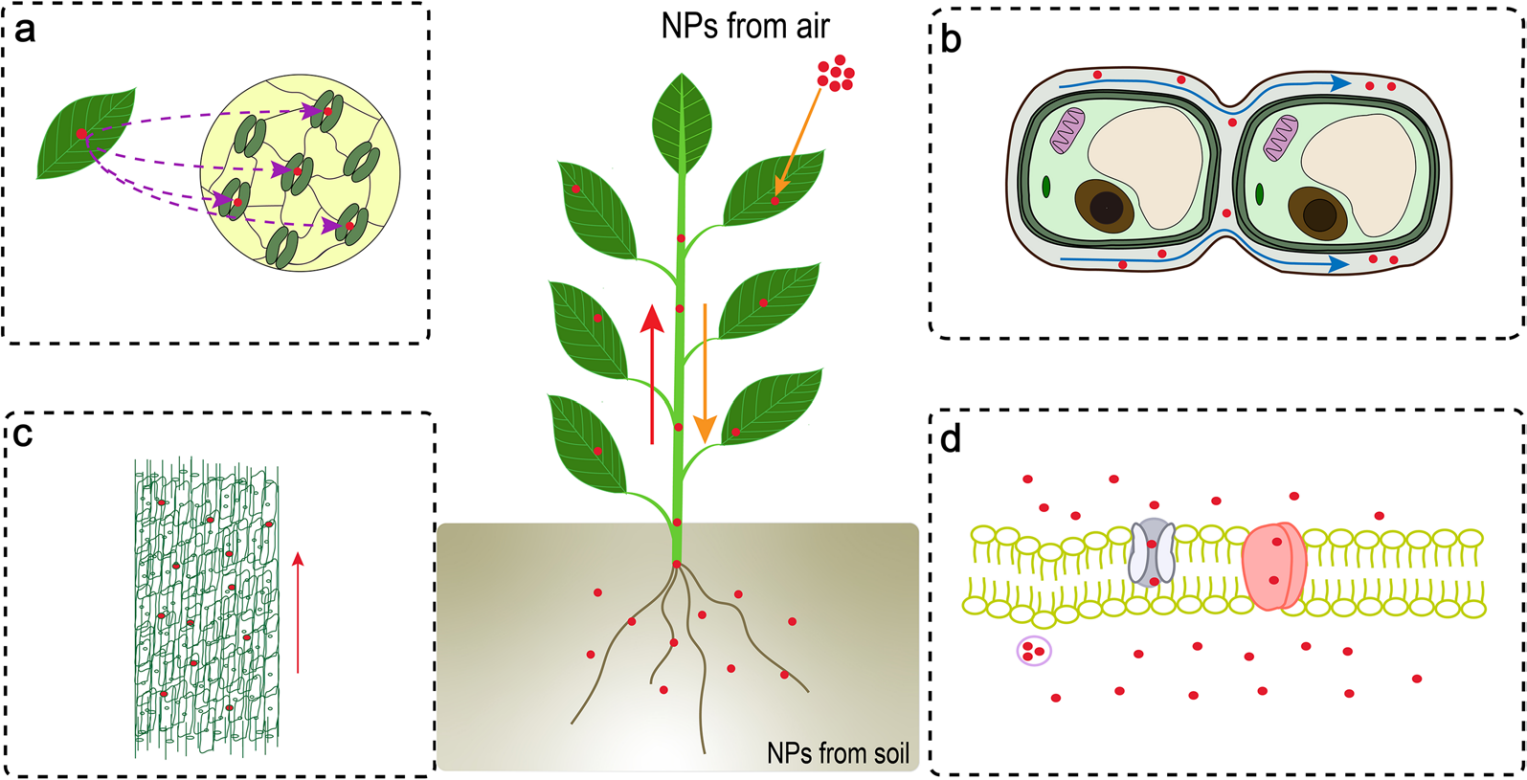
Absorption and transportation mechanism of NPs in plants. **a** NPs are absorbed by stomatal, and transfer from leaves and stems to roots; (**b**) NPs transfer through apoplast pathway; (**c**) NPs enter and transport by xylem and phloem, and transfer from roots to leaves or stems; (**d**) NPs transport through diffusion, endocytosis, and carrier proteins and channels. The red dots represent NPs

Metal NPs discharge from industrial wastewater and electroplating operations can be accumulated in soil and absorbed by plants. Researches have confirmed that metal NPs can enter plant roots and transport to stems and leaves. For instance, 20 nm Ag NPs were detected in mature peanut roots, leaves, and grains, which proved that NPs could be finally transferred to the edible part of peanut, posing a great threat to human health [18]. The Ag NPs could also be absorbed and accumulated in onion cells that are confirmed by TEM (Fig. 2a) [43]. Synchrotron radiation X-ray microanalysis, high-resolution electron microscopy and X-ray near edge absorption spectroscopy are used to locate Au NPs in tobacco. These results suggested that 3.5 nm Au NPs entered plants through roots and penetrated into the vascular system of plants [44]. The transport process of Au NPs (the average diameter is 100 nm, and the aggregates are about 1.25 µm at most) in *Perilla frutescens* was studied by two-photon excitation microscopy and X-ray imaging. The results showed that Au NPs could be ingested by plants through direct penetration and transport through stomatal openings (Fig. 3a). The evidence of translocation of Au NPs from leaf to root confirmed the mechanism of phloem transport [10]. Besides Ag and Au NPs, nano-zerovalent Fe could enter the plants as well. They were absorbed by the roots of *Agrostis capillaris* and *Festuca rubra*, although the proportion of absorption was different because of the diverse species [45].

### Transportation and distribution of metal oxide NPs in plants

Metal oxide NPs have been widely used in biomedicine and catalyst because of their special physical and chemical properties [48]. The dramatic increase in their production, estimated at more than 260,000 tons per year worldwide, has forced attention to their safety [49]. Once released into the soil, metal oxide NPs can be absorbed by plants and caused phytotoxicity. Adams et al. analyzed the wheat root surfaces that exposed to CuO NPs (< 50 nm), and observed the existence of copper signal in the attached particles. The NPs gathered on the surface, causing curl of root hairs and changed in cell structure of root apical meristems, proving that CuO NPs could be transported by wheat roots and had toxic effects [50]. CuO NPs (25 nm ± 1 nm) could also be taken up by wheat roots and accumulated in root cells. TEM analysis showed that the root of wheat had normal ultrastructure. However, the accumulation of electron density was observed in plant cells treated with CuO NPs. Images confirmed that CuO NPs could penetrate the root and accumulate in cells (Fig. 2b) [19]. Similarly, ZnO NPs (10–70 nm) were detected to exist in barley roots at the early growth stage in moderately alkaline soil [51]. In citrus, different Fe_2_O_3_ NPs could enter into cells in different ways. Experiments demonstrated that γ-Fe_2_O_3_ NPs (20 nm) internalized into cells by endocytosis of root, while α-Fe_2_O_3_ NPs (30 nm) internalized mainly through a diapirism process [52]. When studied the transportation of TiO_2_ NPs (80 ± 15 nm) in red bean, the results illustrated that the root accumulation and transportation of TiO_2_ NPs to stem tissues was almost the same. With the extension of exposure time, the accumulation of TiO_2_ NPs in plant tissues increased [53].

### Transportation and distribution of carbon NPs in plants

Carbon NPs are a class of engineering nanomaterials, which have been widely used due to their excellent optical and mechanical properties [54]. Over the past two decades, the synthesis of carbon NPs has developed significantly, and as production and application expand, they are likely to be released into the environment and lead to human exposure [55]. It is important to comprehensively understand the interaction between carbon NPs and plants, especially their absorption and transportation mechanisms. Li et al. used confocal laser scanning microscope to image roots, stems, cotyledons and leaves, and found that CDs were adhered on the roots surface and infiltrated into root vascular tissues. They also confirmed that CDs (under 10 nm) were transported from roots to stems and leaves through vascular system, and ultimately were found in veins rather than mesophyll system, proving that CDs could be transferred and accumulated in plants through vascular system of plants [33]. Researchers also analyzed rice samples and find that C_60_ could be absorbed by rice roots and transfer to stems and rice ears, and aggregates were easily formed in rice tissues. The formation of C_60_ aggregates might be determined by the polarity of C_60_. TEM imaging distinctly showed that C_60_ could penetrate rice cells through cell walls and nuclear membranes, and accumulated in nuclei and vacuoles [32]. Younes et al. treated pepper and eggplant leaf cells with graphene nanosheets (GNS) solution. TEM imaging showed that GNS of both plants were located in plastids, cell walls and intercellular spaces, demonstrating that GNS could be internalized by cells (Fig. 2c, d) [46].

### Transportation and distribution of plastic NPs in plants

Plastic pollution has become a global concern for ecosystem health and biodiversity protection. Large plastics degrade into nano-sized particles due to chemical or mechanical actions [56]. The internalization of plastic NPs by plants may induce phytotoxicity, so understanding how plastic NPs are absorbed and their distribution is the basis of studying the toxic effects on plants. In recent years, the transportation and distribution of PS-NPs in plants have received much attention. It has been reported that PS-NPs (~ 20 nm) entered the maturation zone of rice roots through the promotion of aquaporins and distributed in the intercellular space [57]. Li et al. observed the distribution of PS-NPs in cucumbers and found that the particles were mainly accumulated in the interspace tissues of roots and intercellular space between the ridges in the stem, suggesting that NPs could penetrate into the epidermis of the cucumber root system, reached the cortical parenchyma cells, and transported to the aerial part [42]. In *Arabidopsis thaliana*, the transportation of PS-NPs with different charges (55 ± 7 nm PS-SO_3_H and 71 ± 6 nm PS-NH_2_) was well studied. The results showed that both positively and negatively charged PS-NPs could accumulate in plants. Particles with positive charges were mainly distributed in the mature zone of roots, while negative charges particles were more likely to disperse in apoplast and xylem (Fig. 3b, c) [58]. In addition, Liu et al. observed the absorption of PS-NPs by rice seedlings through fluorescence confocal microscope, and found that the fluorescence of PS-NPs mainly distributed in xylem of rice seedlings (Fig. 2e). Furthermore, the fluorescence intensity of rice seedlings exposed to 80 nm PS-NPs was much higher than that exposed to 1 μm PS-NPs, indicating that small-sized NPs were easier to migrate in plant tissues, and that NPs were more harmful to plants [47].

In conclusion, the accumulation and transportation of NPs in plants may be related to the particle size and charge of NPs. The accumulation of NPs in plants affects agricultural sustainability and food safety, and even threatens human health [59]. It is important to study the relationship between NPs exposure and phytotoxicity. Previous studies have shown that the transportation of NPs can be divided into two pathways: up-to-down and down-to-up. The up-to-down transport route is that when NPs is sprayed on the aboveground, it enters the plant from the stomata of the leaves and transfers in plants through the phloem and xylem [60]. Down-to-up transportation is a process in which NPs are absorbed by plant roots and transferred to stems and leaves. Researchers have attempted to clarify the absorption mechanism of NPs by plant roots, and find that cell-to-cell symbiotic pathway is the main routes of transportation. In addition, there are two barriers that can transfer NPs through symbiotic pathways. One is that NPs penetrate the cell membrane and enter the cytoplasm. The other is that NPs is internalized by cells and transported to adjacent cells by plasmodesmata [61]. The apoplast pathway is considered to be another main pathway for transportation, that is, NPs is generally taken up by the endoderm of roots through apoplast pathway, and then transport to the stems, leaves and fruits of plants through xylem or phloem [62, 63]. It has also been reported that carrier proteins, interconnected channels and endocytosis can also promote the transport of NPs (Fig. 3d) [64]. Hydrophobic interaction is important to complete the endocytosis process. Compared with other NPs, plastic NPs have better hydrophobicity, and may be easier to transport in plants through endocytosis [65, 66].

The absorption and transportation of NPs in plants may be more related to the species of plants, the size of NPs and surface charge. Differences in physiological and metabolic of plants may lead to differences in absorption and transport of NPs. Studies showed that the translocation rate of Ce NPs in dicotyledonous tomato was higher than that in monocotyledonous festuca [67]. Compared with wheat, the root to shoot transport of CeO_2_ NPs in pumpkin was more efficient [68]. The results indicated that plant species may be one of the factors affecting the absorption of NPs. The difference of NPs absorption between monocotyledons and dicotyledons may be due to the different vascular and structural characteristics of taproot system in dicotyledons and fibrous root system in monocotyledons, it might also be due to the greater cation binding capacity in dicotyledons [69]. Particle size of NPs is an important characteristic that affects the absorption of NPs by plants. Smaller NPs may be more easily absorbed by plants due to biological barrier limitations. Generally, in plants, cell wall pore is only 10 nm, plasmodesmata is usually cylindrical with a width of about 40 nm, and aquaporin pore is only about 1 nm, and endocytosis can even pass-through NPs with size of 1 μm [60]. Confocal images of the distribution of PS-NPs with four particle sizes of 100, 300, 500 and 700 nm in cucumber plants suggested that PS-NPs with 100 nm was more easily absorbed by cucumber [70]. TEM and μ-XRF results suggested that TiO_2_ NPs with the size of less than 36 nm accumulated in wheat roots and transported in plants. NPs with the size of 36 ~ 140 nm mainly accumulated in the root system, and there was no translocation in the aerial part. NPs larger than 140 nm did not accumulate in the root system [71]. The above results indicated that the absorption and distribution of NPs in plants may be size-dependent. The size of CDs is usually less than 10 nm, which is smaller than other NPs, and may be more easily absorbed by plants [33]. A comprehensive investigation of diverse plants suggests that the surface charge of NPs is another key factor affecting plant absorption and potential toxicity. Zhu et al. studied the absorption and transport of PS-NPs in wheat roots by confocal microscope. The results showed that compared with carboxyl modified PS-NPs (PS-COOH), amino modified PS-NPs (PS-NH_2_) seemed to have stronger adsorption capacity on the root surface, but PS-COOH had higher translocation capacity [72]. Koelmel et al. found that surface functionalization greatly affected the absorption and transport of Au NPs in rice roots, with negatively charged NPs preferentially transported through the vascular system, while positively charged NPs were more likely to accumulate in the roots [73]. Positively charged NPs were more easily absorbed by the root surface than negatively charged ones. Therefore, the content of positively charged NPs in roots were significantly higher than negative NPs, but compared with negatively charged NPs, the internalization rate and transmission efficiency of positive particles in plants were far lower [74]. The reason might be that the root cap of plants is protected by a mucous layer of marginal cells composed of a layer of negatively charged root exudates, making it easier for positively charged NPs to accumulate in roots [61]. The influence of different factors on the absorption of NPs by plant roots is complicated. In fact, the data on the transportation of NPs in plants are relatively limited at present, further study needs to be carried out to understand their absorption and distribution.

### Effects of NPs to plants

The exposure of NPs leads to their eventual exposure to the soil ecosystem, and then absorbed by edible plants and enter the human body through the food chain [75]. The absorption and distribution of NPs in plants were summarized in the above chapter. After being ingested by plants, NPs interacts with plants, which have positive or negative effects on each growth stage of plants [76]. A better understanding of the effects of NPs on plants can help assess their toxicity (Table 1).

**Table 1:** Effects of NPs on plants

NPs type	NP size (nm)	Plant species	Effects	Refs.
PMMA-NPs	131.3	Lettuce	Decrease of growth, water content and osmotic potential; Reduce stomatal conductance and destroy the reaction center of photosystems; Induced oxidative stress	[17]
PS-NPs	93.6	Lettuce	Reduce the plant biomass, height and leaf area; Electrolyte leakage rate increased significantly; Lead to oxidative stress and damage of antioxidant system; Reduce the content of micronutrients and essential amino acids	[25]
PS-NPs	19 ± 0.16	Rice	Enhance the activities of antioxidant enzymes; Alter phytohormone biosynthesis in anti-stress metabolic pathways	[57]
ZnO NPs	68.14	Coffee	Promote the growth and biomass accumulation	[77]
Fe NPs	52.4 ± 5.1	Pepper	Stimulate the growth of pepper seedlings; Change leaf tissue; Increase chloroplast number and particle accumulation; Regulate vascular bundle development	[78]
Cu NPs	33	Pigeon pea	Increased the height, root length, fresh weight and dry weight of seedlings	[79]
PS-NPs	50	Onion	Root elongation is inhibited at high concentrations	[81]
Ag NPs	20 ± 7, 51 ± 7 and 73 ± 5	*Vicia faba*	Reduce chlorophyll content and photosynthesis; Increase the production of ROS	[83]
Ag NPs	7.5–70	Wheat	Reduce mitotic index; Cause chromosome aberration; Cause nuclear erosion and elongation	[84]
Al_2_O_3_ NPs	0–60	Soybean	Change the root surface structure and destroy root cap; Enhance POD activity	[82]
Ag NPs	22	Cucumber	Decrease the biomass, chlorophyll and carotenoid contents of seedlings; Inhibit photosynthesis; Reduce zinc and iron nutrients contents	[90]
ZnO NPs	20–45	*A. thaliana*	Reduce the length of primary root; Change the contents of major- and micro-nutrients	[91]
Carbon nanodots	3	*A. thaliana*	Root elongation is inhibited; RNA-seq analysis indicates transcriptomics response; The content of metabolites has changed significantly	[92]
Ag NPs	25.3	*Vigna radiata* and *Brassica campestris*	Inhibit seedling growth; Destroy the integrity of vacuoles and cell walls	[95]
Ag NPs and ZnO NPs	11 ± 0.7	Maize and *B. oleracea*	Reduce the size of vacuoles Reduce the turgidity and size of cells	[96]
PS-NPs	160	Wheat	Affect root cell wall and change root anatomical structure	[97]
TiO_2_ NPs	28.5 ± 0.5	*A. thaliana*	Increase antioxidant enzyme activity and lipid peroxidation; Affect the expression level of tocopherol biosynthesis genes	[107]
Cr_2_O_3_ NPs	239.9, 265, 326, 340 and 336	Onion	Improve antioxidant enzyme activity; Reduce mitotic index; Cause chromosome aberration	[111]
PS-NPs	101.7 ± 1.7	Onion	Reduced root length Induced the production of hydroxyl and superoxide radicals Induced chromosome abnormality and nuclear aberration	[113]
Al_2_O_3_, ZnO and Ag NPs	15–60	Soybean	Affect seedling growth; Generate oxidative stress; Affect the protein related to secondary metabolism and cell tissue	[116]
NiO NPs	10–20	Chinese cabbage	Reduce the growth of buds and roots; Reduce the content of chlorophyll and carotenoid; Enhance ROS production and lipid peroxidation level; Cause metabolic and molecular changes	[117]

### Effects of NPs on plant development

**Fig. 4:**
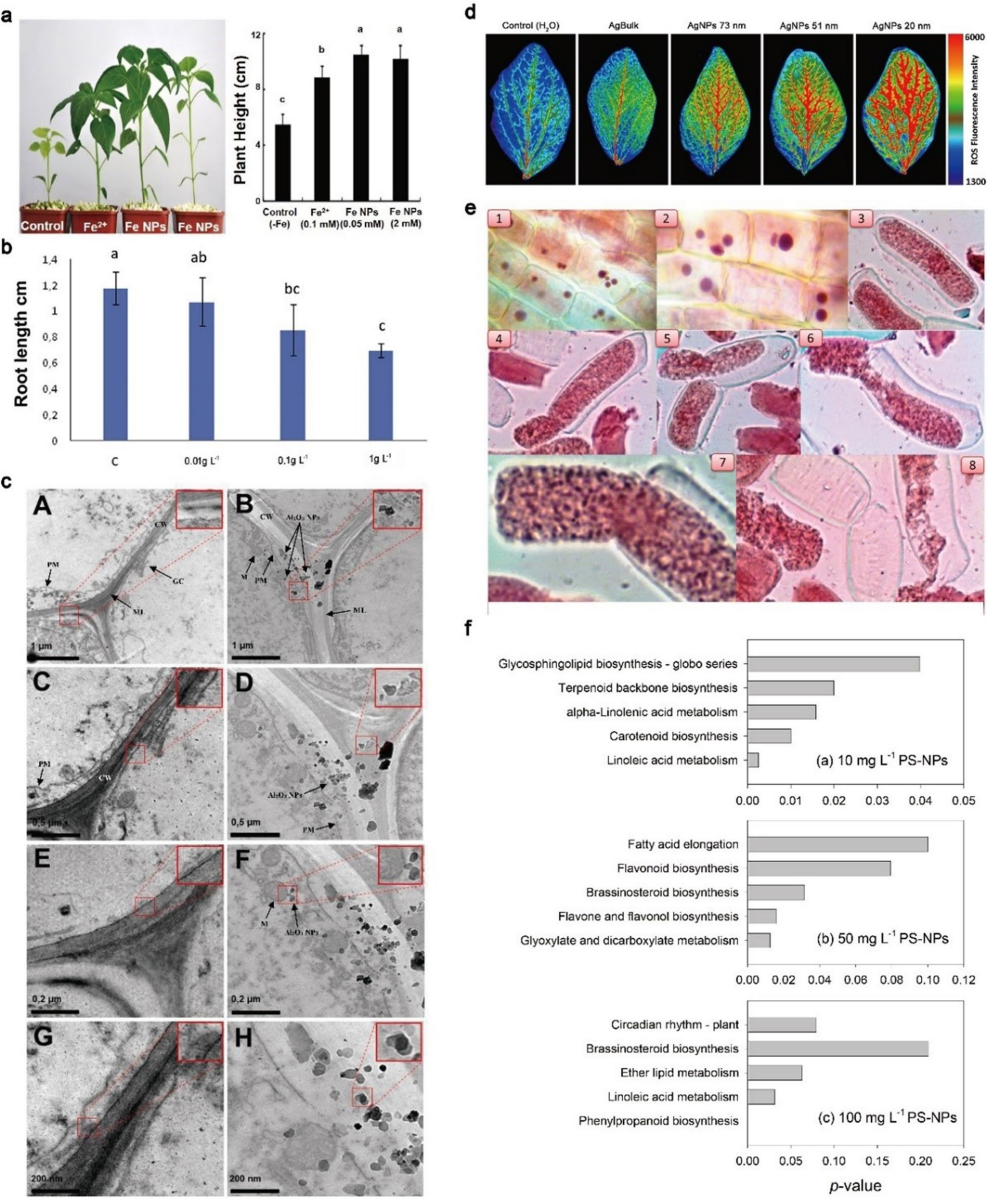
Effects of NPs exposure on plants. **a** Exposure of Fe NPs promoted the growth of pepper seedlings [78]; **b** Plastic NPs exposure inhibited the root elongation of wheat seedlings [81]; (**c**) Exposure of Al_2_O_3_ destroyed the plasma membrane structure of soybean roots (*PM* plasma membrane, *CW* cell wall, *ML* middle lamella, *GC* Golgi complex, *M* mitochondria) [82]; (**d**) The internalization of Ag NPs increased the level of ROS in *Vicia faba* leaves [83]; (**e**) Ag NPs induced chromosome aberration in wheat root tip cells [84]; **f**, Plastic NPs exposure stimulated the transcriptomics response of rice [57]

Researches have suggested that the exposure concentration of various NPs below certain limits may stimulate the growth of plants and seed germination [60]. Spraying 10 mg L^−1^ ZnO NPs on leaves could promote the growth and biomass accumulation of coffee plants. Researchers concluded that this might be due to the increasing of the net photosynthetic rate and thus promoting plant growth [77]. Yuan et al. found that low concentration of Fe NPs could stimulate the growth of pepper seedlings. And microscope analysis suggested that Fe NPs benefited plant growth by changing leaf tissue, increasing chloroplast number and particle accumulation, and regulating vascular bundle development (Fig. 4a) [78]. In the study of the influence of Cu NPs on pigeon pea, it is found that Cu NPs treatment significantly increased the height, root length, fresh weight and dry weight of seedlings. Using handy-plant efficiency analyzer to measure chlorophyll to evaluate photosynthesis, it was concluded that Cu NPs promoted plant growth by enhancing photosynthesis [79]. Results showed that the exposure of low-dose NPs could promote seed germination and growth of different plants, but the mechanism of positive effects of NPs on plants was not illustrated [80].

At present, most studies focus on the harmful effects of NPs on plants, which depend on particle chemical properties, size and reactivity, especially the number of NPs inside the plant or attached to the plant. The negative effect of NPs on plant growth is mainly caused by excessive NPs [60]. Soil ecosystem, especially farmland soil, is the sink of NPs, where the NPs can be inevitably absorbed and accumulated by plants [85]. Their enrichment in plants usually changes physiological processes by reducing photosynthesis and transpiration rate, and eventually affects plant growth [86]. Studies reported that NPs might reduce root and stem length by decreasing germination rate, and influenced plant development by disrupting photosynthesis, inducing oxidative damage, changing the activity of antioxidant enzymes, and unbalancing nutrient composition of edible crops [36, 87]. Giorgetti et al. analyzed the germination rate and root length of onion after 72 h plastic NPs exposure. The results showed that the root length of 0.1 g L^−1^ and 1.0 g L^−1^ plastic NPs treatment decreased in a dose-dependent manner, which confirmed the inhibitory effect of plastic NPs on root elongation at high concentration (Fig. 4b) [81]. The underlying mechanism might be due to the particles attached to the peripheral root tissues, obstructing the absorption of water and reducing the hydraulic conductivity and inhibited transpiration [88, 89]. To study the toxicity of Ag NPs to cucumber growth, the root and stem length, fresh and dry weight of cucumber seedlings were measured after treated with 500 μM and 1000 μM Ag NPs. The results showed that the biomass, chlorophyll and carotenoid content of seedlings all decreased, the photosynthesis was inhibited, and zinc and iron nutrients were reduced after treatment [90]. Researchers studied the toxic effects of ZnO NPs on *A. thaliana*, and found that high concentration (50, 100 and 200 mg L^−1^) of particles led to leaf size reduction and chlorosis, and lateral roots inhibition, which might be caused by the suppression of nutrients absorption in roots, resulting in the decrease of micro-nutrient level [91]. Compared with the control group, the root elongation of *A. thaliana* was significantly reduced by 25%, 40%, 56% and 61% when exposed to CDs with a size of 3 nm at concentrations of 125, 250, 500 and 1000 mg L^−1^, respectively, indicating that the phytotoxicity induced by CDs increased in a dose-dependent manner [92].

The above results demonstrate that NPs can cause phytotoxicity and inhibit the germination and growth of plants by restraining photosynthesis and reducing nutrient content. From the microscopic viewpoint, NPs may penetrate into the plant cell wall and destroy the cell structure.

### Effects of NPs on plant cell structure damage

Studies have shown that the existence of some NPs not only adversely affect the growth of plants, but also destroy the integrity of cells and subcellular organelles, resulting in the loss of cell membrane integrity and mitochondrial function [93].

The effects of Al_2_O_3_ NPs on the cell wall structure of soybeans were analyzed, and the results showed that compared with the control group, the exposure of Al_2_O_3_ NPs could change the root surface at the microscopic level, leading to the formation of cracks near the root tips and the damage of root cap (Fig. 4c) [82]. After being internalized by soybean, Al_2_O_3_ NPs interacted with the mitochondria and chloroplasts, and stimulated the production of reactive oxygen species (ROS), indicating that the occurrence of cell damage might be caused by ROS production [94]. In a study of adverse effects of Ag NPs on *Vigna radiata* and *Brassica campestris*, researchers discovered that Ag NPs could be internalized into cells and destroyed the integrity of vacuoles and cell walls, possibly affecting other organelles as well [95]. Similarly, Tripathi et al. assessed the effects of Ag NPs and ZnO NPs exposure on maize and* B. oleracea*, and found that both NPs could reduce the size of vacuoles, and ultimately reduced the turgidity and size of cells [96]. TEM images showed that exposure to PS-NPs would break chloroplast structure and damage the cell structure of lettuce [25]. The results of Fourier transform infrared spectroscopy (FTIR) and synchrotron computer micro-tomography showed that the exposure of PS-NPs (PS-NPs doped with Pd) might affect the cell wall of wheat roots, thus changing the root anatomical structure [97].

### Toxicity mechanism of NPs

**Fig. 5:**
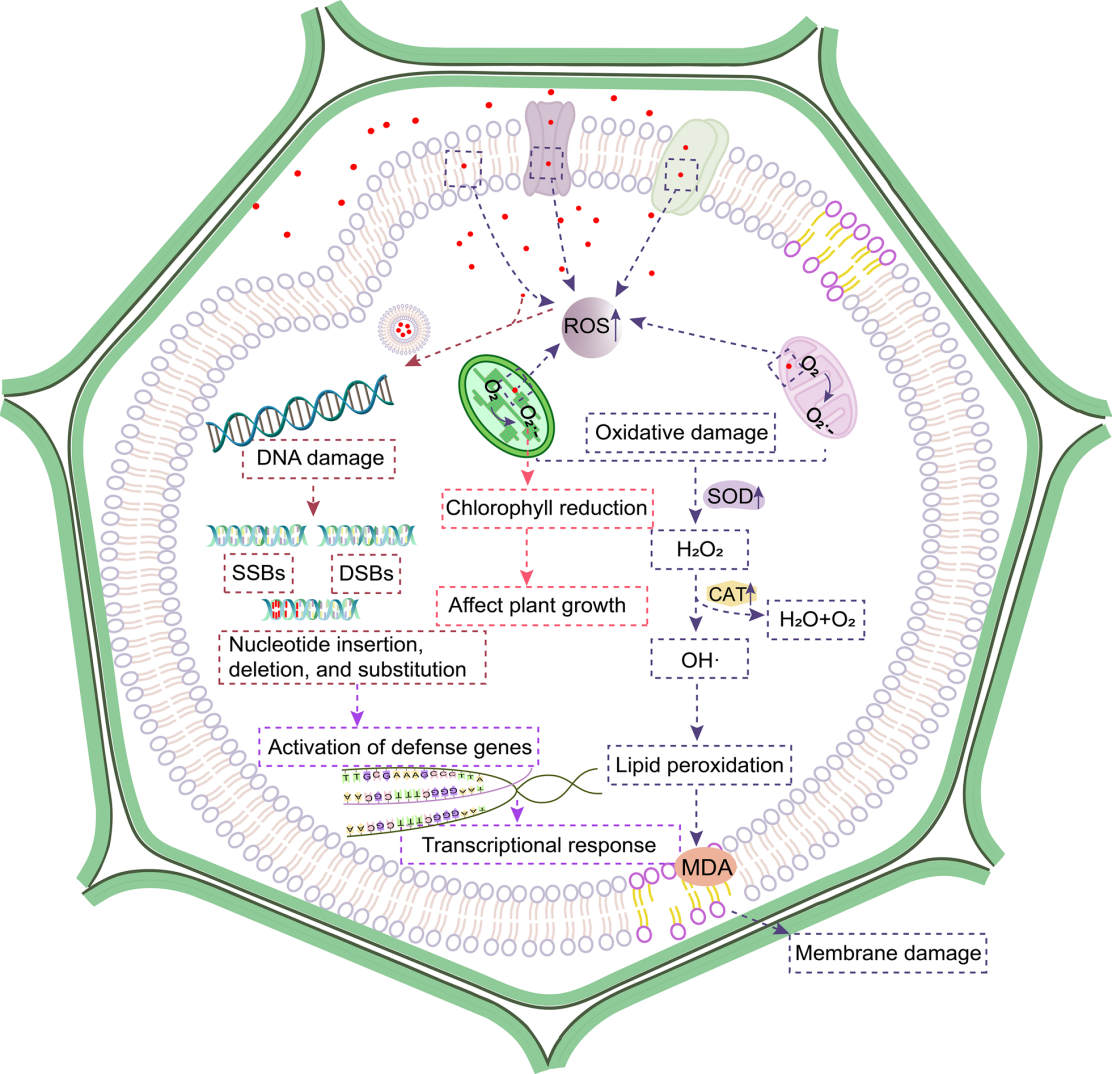
Mechanism of phytotoxicity induced by NPs. *CAT* catalase; *DSBs* double strand breaks, *MDA* malondialdehyde, *ROS* reactive oxygen, *SOD* superoxide dismutase, *SSBs* single-strand breaks. The red dots represent NPs

Some NPs can be transported and accumulated in plants, leading to phytotoxicity. The mechanisms of plant toxicity induced by NPs are summarized as follows: (1) the production of ROS increases during the interaction between NPs and plant, leading to oxidative damage [98]; (2) transcriptional response caused by NPs [99]; (3) the interaction between NPs and DNA or organelles (such as mitochondria), resulting in genotoxicity (Fig. 5) [100].

### Oxidative stress damage

Like other aerobic organisms, plants select ROS as a signal molecule to motivate cellular responses to environmental changes [101]. When enter plant cells, NPs can change the level of ROS, inhibit cell metabolism, destroy the antioxidant system, and affect plant growth. ROS is naturally and continuously produced during metabolism by cell organelles, which includes hydroxyl radical, singlet oxygen, superoxide anion and hydrogen peroxide [102, 103]. ROS plays two vital roles, activating defense signal at low concentration, and aggravating damage at high concentration, leading to oxidative damage of amino acids, lipids and nucleic acids [104]. Lipid peroxidation is an important parameter that indicates the integrity of cell membrane [105]. The production of ROS damages the cell membrane through lipid peroxidation, leading to ion leakage and cell metabolism destruction and cell death. Plants can protect cells and subcellular systems from the cytotoxicity of active oxygen radicals by antioxidant enzymes and low molecular weight antioxidants [106]. Therefore, most of the investigates on oxidative damage of plants caused by NPs focus on the determination of ROS or antioxidant content and antioxidant enzyme activity. The existence of Ag NPs in bean leaves activated the generation of ROS. The data showed that the ROS production was increased with the decrease of particle diameters since smaller NPs had higher specific surface area and present stronger cytotoxicity (Fig. 4d) [83]. Under high light conditions, the existence of TiO_2_ NPs led to the increase of tocopherol content and superoxide dismutase (SOD) activity, with decreased catalase (CAT) activity. This might be related to the photoactivation of particles and the increase of ROS production, indicating that higher NPs concentrations cause membrane lipid peroxidation [107]. The application of polymethyl methacrylate nanoplastics (PMMA-NPs) caused the oxidative stress of lettuce, and the activity of antioxidant enzymes increased, but the increase of defense activity did not reduce the attack of free radicals. Compared with the control, it still showed an increase in the level of active oxygen [17]. Recently, scientists confirmed that with the increase of plastic NPs concentration, the activities of several representative antioxidant enzymes in rice roots were enhanced, suggesting that plants could stimulate defense responses and remove excesses accumulation of ROS [57].

ROS is usually formed by the inevitable leakage of electrons to O_2_ during electron transport activities in chloroplasts, mitochondria and plasma membranes [108]. Excessive accumulation of ROS in plant cells will destroy the steady state. When the ROS level exceeds the defense mechanism, the cells will be in an ‘‘oxidative stres’’ state, causing infinite damage to protein, nucleic acids and lipids in cells membranes, and inducing the expression of relative defense genes to respond to these injuries [103].

### Genotoxic

Environmental discharge of NPs leads to their accumulation at diverse nutritional levels. Plants are highly susceptible to nanotoxicity and can absorb NPs [109]. For this reason, plants are regarded as extraordinary genetic models for sifting and monitoring environmental toxic compounds [110]. NPs can interact with biological macromolecules (nucleus, cytoplasmic components, or lipids) to exert cytotoxic and genotoxic effects in plants, such as reducing mitotic index, and enhancing micronucleus and chromosome aberration index [40]. Researchers evaluated the effects of Ag NPs on wheat chromosome aberrations and cell division, and found that the treated root tip cells showed various types of chromosome aberrations, such as multicore, incorrect orientation at metaphase, chromosome breakage, metaphase plate deformation, spindle dysfunction, and lagging chromosomes, indicating that the root tip cells of wheat internalized Ag NPs easily, and the particles could interfere with the normal function of the cells to inhibit the synthesis of DNA in the S phase of interphase (Fig. 4e) [84]. The cytogenetic effects of Cr_2_O_3_ NPs on onion root cells were analyzed to find that the mitotic index had a significant decrease. There were also chromosome aberrations under different exposure concentrations [111]. According to previous research, the internalization of Cr_2_O_3_ NPs increased the production of SOD to counteract oxidative stress, leading to observed DNA damage [112]. PS-NPs exposure could significantly reduce the mitotic index of onion, and different types of chromosome aberrations (including aggregation, lagging chromosomes, etc.), spindle deformation and nuclear abnormality were observed, which destroyed the stability of the genome, indicating the genotoxicity induced by PS-NPs [113].

In general, the mechanism of genotoxicity can be divided into direct genotoxicity or indirect genotoxicity. Direct genotoxicity comes from the physical interaction between NPs and DNA, while the indirect genotoxicity may be due to the decrease of DNA repair or the increase of ROS content [114].

### Transcriptomic response

Studies have proved that NPs cause adverse effects on plants because of their persistent behavior. Transcriptomics provide new insights into the process of interaction by establishing the relationship between gene expression and cell metabolism [115]. For example, Hossain et al. validated that exposure to Ag NPs could down-regulate the expression of subtilisin family protein in soybean leaves, which resultantly attributed to the decrease of seedling stem length [116]. NiO NPs could increase the transcription level of antioxidant enzymes in Chinese cabbage seedlings, as well as the anthocyanin and proline biosynthesis related genes in response to oxidative stress [117]. The elevated plastic NPs exposure in rice significantly alters phytohormone biosynthesis in anti-stress metabolic pathways, demonstrating its important physiological role in plant stress response (Fig. 4f) [57]. Transcriptome analysis revealed that NPs exposure significantly changed the processes of carbon metabolism, amino acid biosynthesis and plant hormone signal transduction in wheat, and revealed the phytotoxicity mechanism induced by plastic NPs [27].

The comprehensive response of plants to specific NPs stress is manifested by the differential expression of genes, which participate in a series of biological reactions, such as redox, detoxification, hormone signal pathways and signal transduction. Transcriptional studies have provided evidence of stress induced by NPs at the genetic level, but the research on transcriptional response induced by NPs is not sufficient at present, further actions need to be taken to strive for greater progress.

### Summary and future perspectives

Due to the unique physical and chemical properties, NPs are widely used in different fields. Previous studies mainly focus on the effects of NPs on marine organism. In recent years, more attention has been paid to the toxicity of NPs to terrestrial plants. After being absorbed and internalized by plants, NPs can affect the seed germination and root elongation, depending on the composition, concentration, size, physical and chemical properties of NPs, and also the species of plants [118]. In this paper, the absorption, accumulation and transportation of NPs in terrestrial plants as well as the effects induced by NPs are reviewed. Because of the different sizes and properties, NPs can be internalized by plants in different ways, and they accumulate and migrate, thereby affect the seed germination and plant growth [119]. NPs can promote the growth and seed germination of plants by promoting photosynthesis and increasing the size of xylem and phloem [77, 120]. NPs can also inhibit the growth of plants, destroy the cellular structure of plants, resulting in the cell wall integrity destruction, cell membrane damage, and cytoplasm contraction [95, 121]. It mainly depends on the exposure concentration of NPs. Higher concentrations of NPs are likely to cause plant damage, and many kinds of NPs have been proved that lower concentrations can promote plant growth. In addition, the toxic effect of heavy metals and the adsorption of pollutants by carbon NPs and plastic NPs might also be the reasons for the negative impact on plant growth [122, 123]. The exposure of NPs to plants produces cytotoxicity, increase the content of ROS, and result in oxidative stress, leading to the increase of antioxidant enzyme activity and antioxidant content [87]. Meanwhile, ROS also acts on cell membranes and mitochondria, causing damage to cell membranes and mitochondria [93]. Oxidative stress may indirectly lead to genotoxicity, such as chromosome aberration and micronucleus formation that change the expression of genes and the level of biological components in plants [124]. In addition, NPs release toxic substances to exposed media, such as metal ions into plants, attributing to the phytotoxicity of NPs [125]. Although the impacts of NPs on terrestrial plants have gradually attracted the attention of researchers, the understanding about the mechanism of phytotoxicity induced by NPs is still limited. Most of the studies are carried out in the early development stage of plants rather than the whole life cycle. Therefore, it is of great significance to conduct long-term exposure experiments of NPs, deeply explore their mechanism of phytotoxicity and investigate the impact of NPs on the environment and human health.

## Data Availability

The figures display (Figs. 1, 2, 4) during this review article are available in the Elsevier and Springer Nature repository, and detailed information are shown in the reference list. The figures display (Figures 3, 5 and TOC) during this review article are available from the corresponding author on reasonable request.
